# Purpura Annularis Telangiectodes of Majocchi Following a Booster Dose of the Pfizer-BioNTech COVID-19 Vaccine: Coincidence or Correlation?

**DOI:** 10.7759/cureus.78336

**Published:** 2025-02-01

**Authors:** Amal Al Salmi, Abdullah Al Shibli, Maimuna Al-Saadi

**Affiliations:** 1 Dermatology, Al Buraimi Hospital, Al Buraimi, OMN; 2 Family Medicine, Oman Medical Specialty Board, Muscat, OMN; 3 Pathology, Khoula Hospital, Muscat, OMN

**Keywords:** covid-19 vaccine, pfizer-biontech vaccine, pigmented purpuric dermatosis, purpura annularis telangiectodes of majocchi, side effects

## Abstract

Following the widespread distribution of COVID-19 vaccines, a range of side effects have been reported globally. These effects range from mild reactions, such as injection site discomfort and fever, to rarer events like allergic responses. While most side effects have been mild and transient, their occurrence has prompted further investigation into their frequency, severity, and underlying mechanisms. Despite the generally favorable safety profile of current vaccines, ongoing research remains essential to ensuring their continued safety and effectiveness across diverse populations. In this paper, we present the case of a 25-year-old female diagnosed with purpura annularis telangiectodes of Majocchi following a booster dose of the Pfizer-BioNTech COVID-19 mRNA vaccine. Whether this represents a causal association or a coincidental occurrence warrants further investigation.

## Introduction

Pigmented purpuric dermatosis (PPD) is a broad term encompassing several subtypes of chronic purpuric skin eruptions, characterized by endothelial damage, erythrocyte extravasation, and hemosiderin deposition in the skin. Despite sharing similar histopathological features, these subtypes are primarily distinguished by their clinical presentation [1].

Purpura annularis telangiectodes of Majocchi is a variant of PPD that predominantly affects children and young females [2]. It typically begins on the lower extremities as symmetrical, annular, reddish-brown macules with centrifugal spread, although it may also extend to the trunk and arms [1,2]. While the condition is generally asymptomatic, some cases present with mild pain, itching, or a burning sensation.

The exact etiology of PPD remains unclear, but factors such as capillary fragility, physical activity, venous hypertension, and infections are thought to play a role in its pathogenesis [3]. Recently, PPD, including the Majocchi variant, has been reported as a potential cutaneous reaction following COVID-19 vaccination. Although rare, these manifestations underscore the need for further investigation into vaccine-related dermatological responses.

## Case presentation

**Figure 1:**
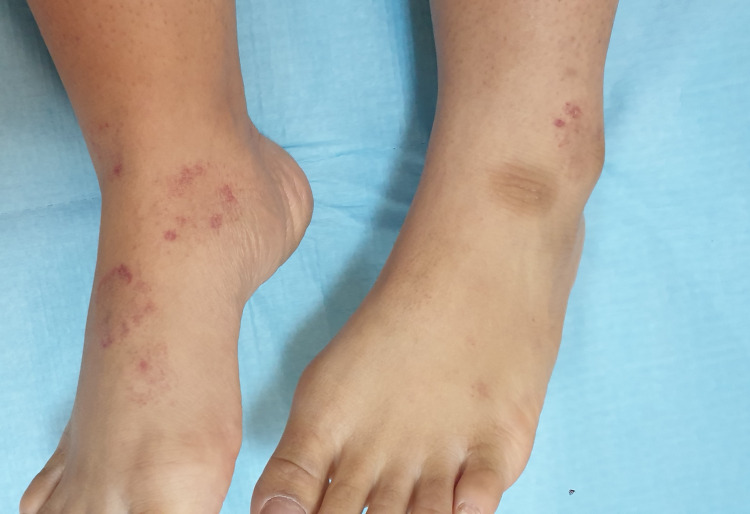
Non-blanchable annular and arcuate erythematous patches on the dorsum of the feet

A 25-year-old female with no known medical issues presented to the dermatology department with a four-month history of erythematous rashes, primarily on the dorsum of her feet and, to a lesser extent, on her distal legs. She also reported a few similar lesions on both thighs and forearms, which had resolved spontaneously prior to her visit. While she did not experience any symptoms associated with these rashes, she was bothered by their presence. She denied any history of preceding infections, vigorous exercise, or medication use. She had received a booster dose of the Pfizer-BioNTech COVID-19 vaccine less than a month before the rash appeared. Physical examination revealed multiple annular to arcuate erythematous patches on the dorsum of her feet, which were non-blanchable (Figure 1).

Initial laboratory tests conducted in primary care were within normal limits and included a CBC, coagulation profile, and liver and renal function tests (Table 1).

**Table 1:** Baseline laboratory investigations of the patient

Lab tests	Patient results	Normal range
CBC		
Hemoglobin	12.2 g/dL	11-14.5g/dL
Platelet count	225 × 10³/µL	150-450 × 10³/µL
White blood cells	5.02 × 10³/µL	2.4-9.5 × 10³/µL
Coagulation profile		
Prothrombin time	10.8 seconds	10.0-13.6 seconds
Activated partial thromboplastin time	27.2 seconds	22.6-30.6 seconds
Liver function tests		
Total bilirubin	6.4 µmol/L	0-20 µmol/L
Alanine transaminase	11.6 U/L	0-40 U/L
Aspartate transferase	14.8 U/L	5-35 U/L
Renal function tests		
Urea	3.6 mmol/L	2.5-6.7 mmol/L
Creatinine	61 µmol/L	45-90 µmol/L
Sodium	138 mmol/L	135-145 mmol/L
Potassium	4.28 mmol/L	3.5-5.5 mmol/L

**Figure 2:**
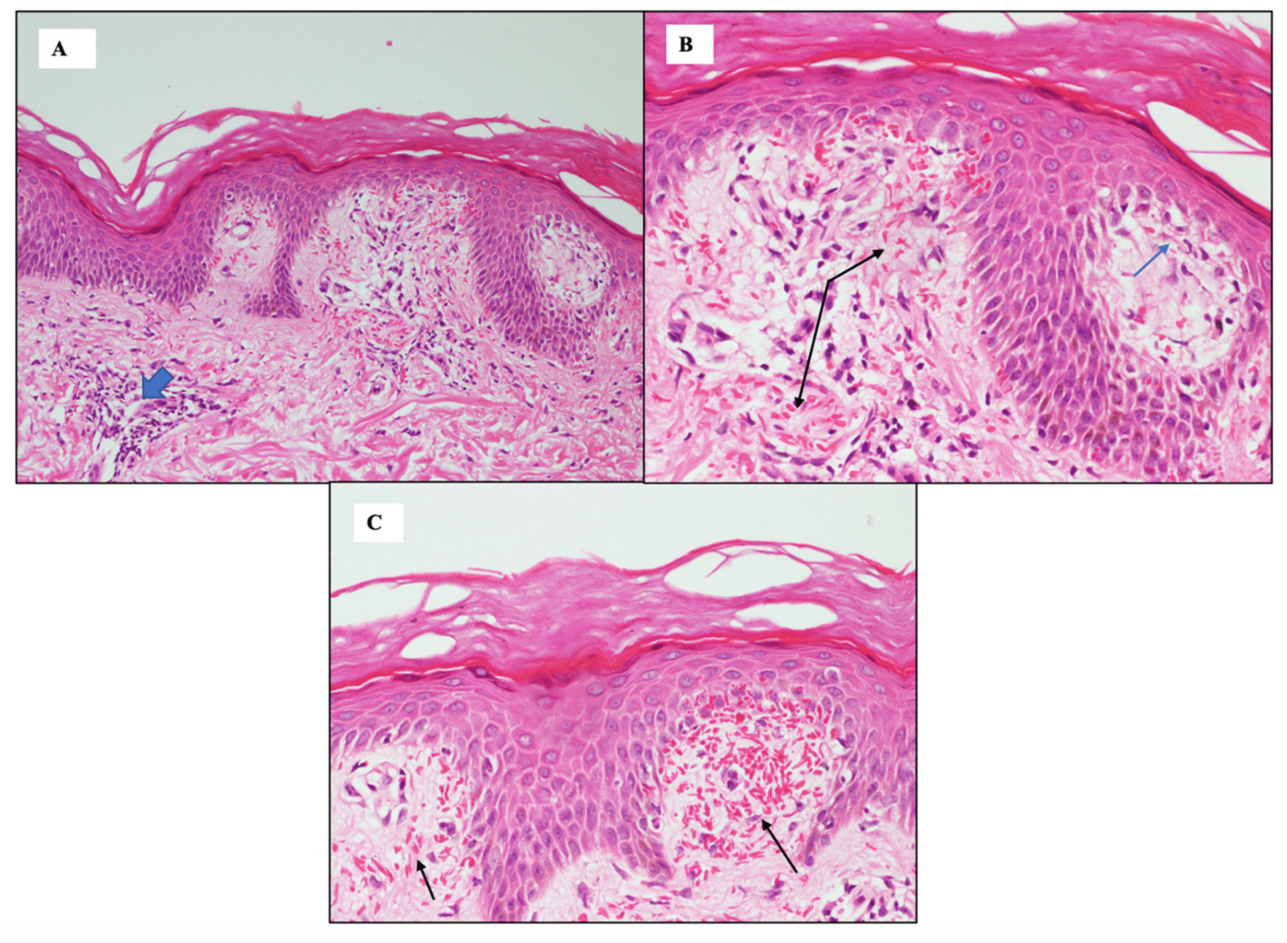
(A) H&E stain (20×) showing hyperkeratosis and mild chronic inflammation in the dermis (thick blue arrow). (B) H&E stain (40×) revealing hyperkeratosis, focal parakeratosis (top right), and focal interface change (blue arrow), along with red cell extravasation (black arrows). (C) H&E stain (40×) highlighting another field with prominent red cell extravasation (black arrows)

We performed a skin biopsy on one of the lesions on the left foot. Histopathological examination revealed hyperkeratosis, focal parakeratosis, and mild basal cell hyperpigmentation in the epidermis. In the dermis, prominent red cell extravasations were observed in the papillary dermis, along with a mild perivascular chronic inflammatory infiltrate in the superficial dermis and evidence of focal interface changes, although no band-like or dense lymphoid infiltrate was present. Periodic acid-Schiff staining revealed no microorganisms. Based on the clinical presentation and histopathological findings, the patient was diagnosed with purpura annularis telangiectodes of Majocchi (Figure 2A-2C).

**Figure 3:**
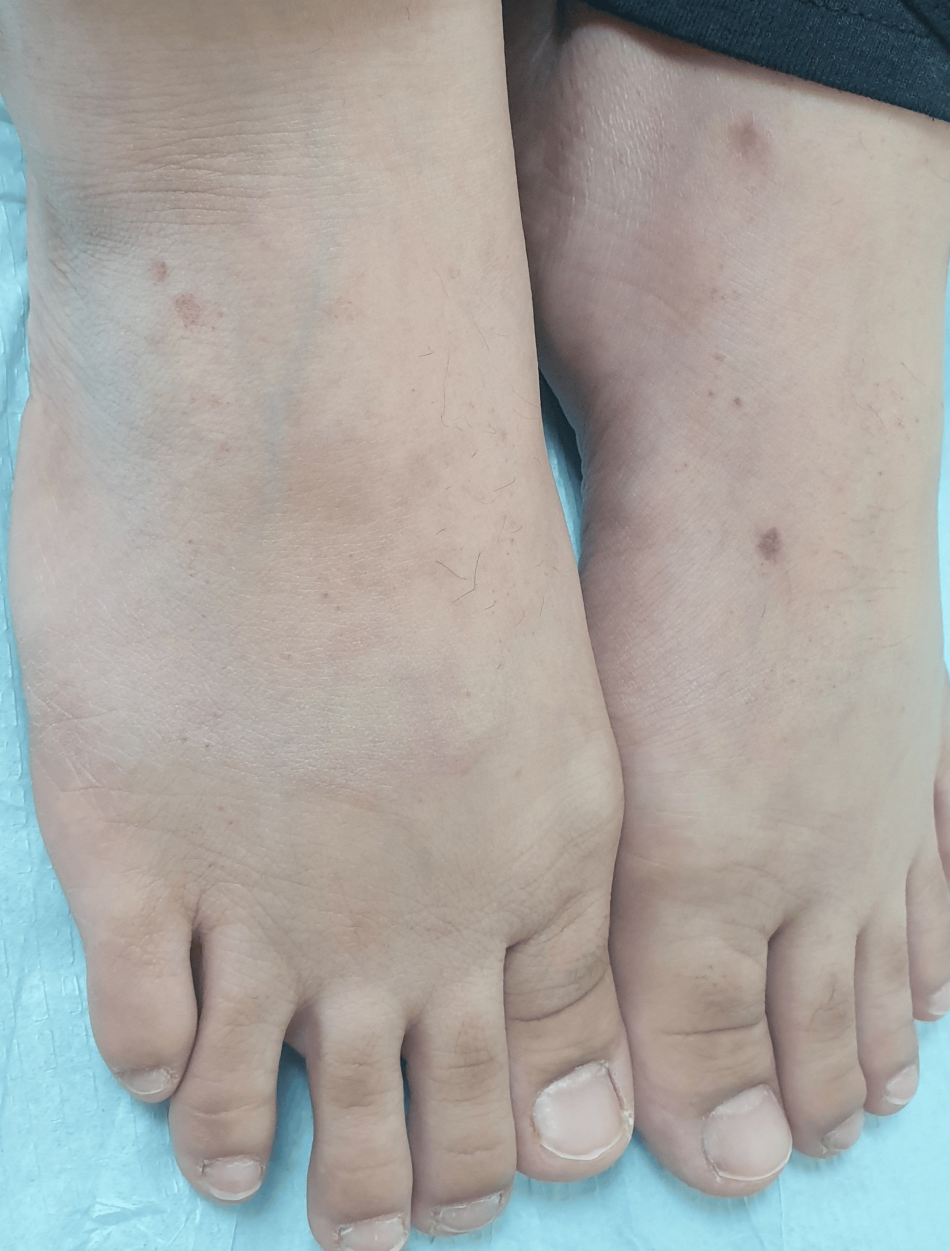
New eruptions and post-inflammatory hyperpigmentation of healed lesions on the dorsum of the feet at the three-month follow-up

The patient was prescribed topical clobetasol propionate cream 0.05% for two weeks, followed by a switch to mometasone furoate cream 0.1%. Most of the rash regressed within six weeks, leaving only mild post-inflammatory hyperpigmentation and a small area of fading petechial rashes. To aid in clearing the hyperpigmentation, the patient was given hydroquinone 4% and continued on mometasone 0.1%. After three months of follow-up, the patient still had a few new eruptions on the dorsum of both feet (Figure 3). Consequently, a short course of oral prednisolone 30 mg was prescribed, along with topical clobetasol propionate cream 0.05% for seven days, followed by a shift to mometasone furoate cream 0.1%. During the six-month follow-up period, only a few scattered lesions were observed, which cleared within two to three days with the application of topical steroids. At the eight-month follow-up, no new eruptions were reported, and the patient was subsequently discharged from the clinic.

## Discussion

In this article, we presented the case of an adult female patient who developed purpura annularis telangiectodes of Majocchi following the third dose of the Pfizer-BioNTech COVID-19 vaccine, administered less than one month prior. The close timing between the vaccine and the onset of skin symptoms raises the possibility of a connection between the two events. Our hypothesis is further supported by the absence of other clinical symptoms and a lack of relevant medical history.

Globally, the prevalence of cutaneous adverse reactions following COVID-19 vaccination is approximately 3.8% [4]. Numerous studies have documented various cutaneous side effects associated with COVID-19 vaccines, ranging from mild reactions, such as injection-site erythema and morbilliform rashes, to more severe cases like urticaria, vasculitis, and exacerbations of preexisting dermatological conditions [4,5]. Despite these reports, only a few cases of purpura annularis telangiectodes of Majocchi following mRNA COVID-19 vaccination have been documented [6-8]. One reported case involved unusually widespread rashes of purpura annularis telangiectodes of Majocchi affecting the trunk and limbs [8], while our case presented with more localized rashes confined to the limbs.

Additionally, other forms of PPD following COVID-19 vaccination have been reported, with the majority of these cases following the Pfizer-BioNTech COVID-19 mRNA vaccine [6-9].

Although the pathogenesis of PPD following COVID-19 vaccination remains unclear, cell-mediated immunity is believed to play a significant role. The Pfizer-BioNTech COVID-19 vaccine, which uses mRNA to produce SARS-CoV-2 spike proteins, may trigger capillaritis through immune dysregulation, leading to endothelial damage and resulting in PPD symptoms. Research suggests that the appearance of new skin lesions may be linked to a delayed hypersensitivity reaction or a T-cell-mediated response, triggered by the molecular similarity between the virus and skin cells [10].

Thus, reporting such reactions highlights the potential for COVID-19 vaccines, particularly mRNA vaccines, to trigger certain dermatologic conditions, including PPD. However, further research is necessary to establish a clear causal relationship and to better understand the mechanisms underlying these rare reactions.

## Conclusions

This case report suggests a potential association between the Pfizer-BioNTech COVID-19 mRNA vaccine and the development of purpura annularis telangiectodes of Majocchi. The close temporal relationship between vaccination and the onset of skin symptoms, combined with the absence of other underlying causes, strengthens the hypothesis of a vaccine-related trigger. While cutaneous reactions to COVID-19 vaccines are generally rare, the increasing number of reported cases of purpura and other dermatologic conditions highlights the need for further investigation. Understanding the pathogenesis, particularly the role of cell-mediated immunity and immune dysregulation, is critical to establishing a clear causal link. Continued research is necessary to deepen our understanding of these reactions and their underlying mechanisms.
